# Immunoglobulin Response and Prognostic Factors in Repeated SARS-CoV-2 Positive Patients: A Systematic Review and Meta-Analysis

**DOI:** 10.3390/v13050809

**Published:** 2021-04-30

**Authors:** Fanni Dembrovszky, Szilárd Váncsa, Nelli Farkas, Bálint Erőss, Lajos Szakó, Brigitta Teutsch, Stefania Bunduc, Rita Nagy, Dóra Dohos, Szabolcs Kiss, Andrea Párniczky, Zsófia Vinkó, Zoltán Péterfi, Péter Hegyi

**Affiliations:** 1 Institute for Translational Medicine, Medical School, University of Pécs, 7624 Pécs, Hungary; dembrovszky.f@gmail.com (F.D.); vancsaszilard@gmail.com (S.V.); farkas.nelli@gmail.com (N.F.); dr.eross.balint@gmail.com (B.E.); szaklaj@gmail.com (L.S.); teutschbrigitta@gmail.com (B.T.); stfnbndc@gmail.com (S.B.); nagyrita003@gmail.com (R.N.); dohos.dora@gmail.com (D.D.); kissszabolcs1995@gmail.com (S.K.); andrea.parniczky@gmail.com (A.P.); vinkozsofi@gmail.com (Z.V.); 2 János Szentágothai Research Center, University of Pécs, 7624 Pécs, Hungary; 3 Institute of Bioanalysis, Medical School, University of Pécs, 7624 Pécs, Hungary; 4 Gastroenterology, Hepatology and Liver Transplant Department, Fundeni Clinical Institute, Bucharest 022328, Romania; 5 Heim Pál Children’s Hospital, 1089 Budapest, Hungary; 6 Doctoral School of Clinical Medicine, University of Szeged, 6720 Szeged, Hungary; 7 Division of Infectious Diseases, First Department of Medicine, Medical School, University of Pécs, 7623 Pécs, Hungary

**Keywords:** COVID-19, meta-analysis, prognosis, repositivity, SARS-CoV-2

## Abstract

With repeated positivity being an undiscovered and major concern, we aimed to evaluate which prognostic factors may impact repeated SARS-CoV-2 positivity (RSP) and their association with immunoglobulin detectability among recovered patients. A systematic literature search was performed on 5 April 2021. Cohort studies with risk factors for repeated RSP or information about the immunoglobulin response (immunoglobulin M (IgM) and/or immunoglobulin G (IgG)) were included in this analysis. The main examined risk factors were severity of the initial infection, body mass index (BMI), length of hospitalization (LOH), age, and gender, for which we pooled mean differences and odds ratios (ORs). Thirty-four cohort studies (*N* = 9269) were included in our analysis. We found that increased RSP rate might be associated with IgG positivity; IgG presence was higher in RSP patients (OR: 1.72, CI: 0.87–3.41, *p* = 0.117). Among the examined risk factors, only mild initial disease course showed a significant association with RSP (OR: 0.3, CI: 0.14–0.67, *p* = 0.003). Age, male gender, BMI, LOH, and severity of the first episode do not seem to be linked with repeated positivity. However, further prospective follow-up studies focusing on this topic are required.

## 1. Introduction

More than one year after the outbreak of the severe acute respiratory syndrome coronavirus 2 (SARS-CoV-2) pandemic, the number of confirmed infections exceeds 100 million and it has caused over 2 million deaths [1,2]. Some viral infections can lead to life-long immunity, such as morbilli, mumps and rubella, with low antigenic variability [3]. However, a single first episode of coronavirus disease 2019 (COVID-19) might not prevent a recurrence, and previous reports and a meta-analysis stipulated the possibility of reinfection [4,5,6,7].

A study found that recurring SARS-CoV-2 polymerase chain reaction (PCR) positivity might occur in 2.4% to 69.2% of the patients [2]. However, reinfection is not defined by an international consensus. Repeated PCR positivity might result from relapse of the disease, false positivity, or reinfection. Positivity may also be caused by the remaining viral fragments in the case of a PCR with a high cycle threshold [8].

The literature is contradictory, considering both the potential existence of reinfection and patients’ infectivity with repeated PCR positivity. A narrative review and a Korean study suggest that repeated positivity might not be associated with infectious state [2]. Furthermore, a study found that reinfection cannot occur in Rhesus macaques [9]. The meta-analysis of Arafkas et al. concluded that the persistence of test positivity is caused by a prolonged disease course rather than reinfection [10]. On the other hand, among the several case reports on patients with possible reinfection, very few differentiated between the strains of SARS-CoV-2.

Considering reinfection, one of the main questions is whether specific antibodies provide immunity against a second episode. Anti-SARS-COV-2 Immunoglobulin G (IgG) positivity was found in more than 95% of the patients after the recovery from the infection [11]. Despite this, there is still no consensus whether they are protective for the second episode of disease and serology data for the repositive cases are lacking. Based on the literature RSP, an earlier confirmed COVID-19 patient produces a repeatedly positive RS-PCR test after two consecutive negative results (with at least a 24 h sampling interval) of the nucleic acid tests of respiratory pathogens during the follow-up period [12]. Lumley et al. investigated the relationship between antibody positivity and the risk of SARS-CoV-2 reinfection in a prospective cohort study and showed that the presence of IgG antibodies reduces the risk of further infection [13].

While clear definitions are missing considering the meaning of a PCR positivity after a negative test, a recurring disease’s clinical importance should be highlighted. The probability of disease recurrence in COVID-19 patients is a significant concern, and the relevance of PCR repositivity needs to be better understood in terms of causes and predisposing factors. A Danish population-level observational study examined the factors that protect against reinfection and found that 80% of younger people (<65 years) are protected compared to 47% of older people (>65 years) [14]. However, we are not yet aware of prognostic factors potentially influencing the recurrence of positive results of PCR.

In this meta-analysis of cohort studies, we aimed to assess the prognostic factors for repeated SARS-CoV-2 positive episodes (RSPs) and the prognostic value of immunoglobulin positivity (seroconversion).

## 2. Materials and Methods

The study protocol of the systematic review and meta-analysis was registered to the International Prospective Register of Systematic Reviews (PROSPERO) with the registration number CRD42021233618 (see https://www.crd.york.ac.uk/prospero, accessed on 29 March 2021). It was performed adhering to the guidelines established by Preferred Reporting Items for Systematic Reviews and Meta-Analyses (PRISMA) [15]. Protocol deviation did not occur.

### 2.1. Search

A systematic literature search was conducted in Cochrane Central Register of Controlled Trials (CENTRAL), Embase and MEDLINE (via PubMed) for studies published from inception to 5 April 2021. The following search key was applied: ((“covid 19”) OR (“coronavirus”) OR (“2019 nCoV”) OR (“SARS-cov-2”)) AND ((reinfection) OR (“second episode”) OR (“second infection”) OR (reactivation) OR (recurrence) or (relapse) OR (re-positive) OR (re-detectable) OR (retest-positive) OR (repeated infection)). No language or other restrictions were imposed.

### 2.2. Selection and Eligibility

Duplicate removal of yielded articles was performed by a reference management program (EndNote X9, Clarivate Analytics, Philadelphia, PA, USA). Two independent researchers (BT, SB) followed the Cochrane Handbook’s recommendation [16] and simultaneously screened the titles, abstracts, and full texts of the included studies based on predetermined criteria. Cohort studies were eligible for our meta-analysis where authors reported data of patients characterized with any of the terms included in our search key, referring to an RSP. We included only those cohort studies in which any of the characteristics (age, gender, the severity of the initial infection, body mass index, length of hospital stay, antibody presence) of RSP versus non-RSP patients were compared. In case of any disagreement, a consensus was reached after discussion with a third author.

### 2.3. Data Collection

Two review authors extracted the data independently (BT, SB) into a pre-defined Excel datasheet (Office 365, Microsoft, Redmond, WA, USA). An independent third party (FD) settled any discrepancies. The following data were collected from each study: first author, digital object identifier (DOI), study site (country), study design, study period, patient follow-up period, definitions regarding examined patients and events, hospital discharge criteria, demographic data of the whole study population and the repeatedly positive and control groups, days between first and second SARS-CoV-2 positive tests. Data regarding age and sex of the patients, the severity of COVID-19, body mass index (BMI), length of hospitalization (LOH), IgM, and IgG positivity were extracted for both RSP and non-RSP groups.

### 2.4. Risk of Bias and Quality Assessment

Based on the recommendation of the Cochrane Prognosis Methods Group [17], the Quality in Prognostic Studies (QUIPS) tool [18] was applied separately by two authors (RN, DD) to assess the methodological quality of the included studies. Any disagreement was resolved by arbitration by a third investigator.

### 2.5. Statistical Analysis

All meta-analytical calculations were performed by Stata v15.1 software (Stata Corp LLC, College Station, TX, USA). In the case of dichotomous outcomes, odds ratios (ORs) with their 95% confidence intervals (CIs) and weighted mean differences (WMDs) with 95% CIs were calculated for continuous outcomes. Pooled estimates were calculated with the random-effects model using the DerSimonian–Laird estimator [19]. A *p*-value of less than 0.05 was considered a statistically significant result.

Statistical heterogeneity was analyzed using the I^2^ and χ^2^ tests. I^2^ values, representing the magnitude of heterogeneity, were interpreted as moderate (30–60%), substantial (50–90%), or considerable (75–100%). The *p*-value of less than 0.10 was defined as indicating significant heterogeneity [20]. Publication bias was assessed by Egger’s test and by visual inspection of funnel plot asymmetry (alpha = 0.1) when at least ten studies were available.

## 3. Results

### 3.1. Search and Selection

**Figure 1:**
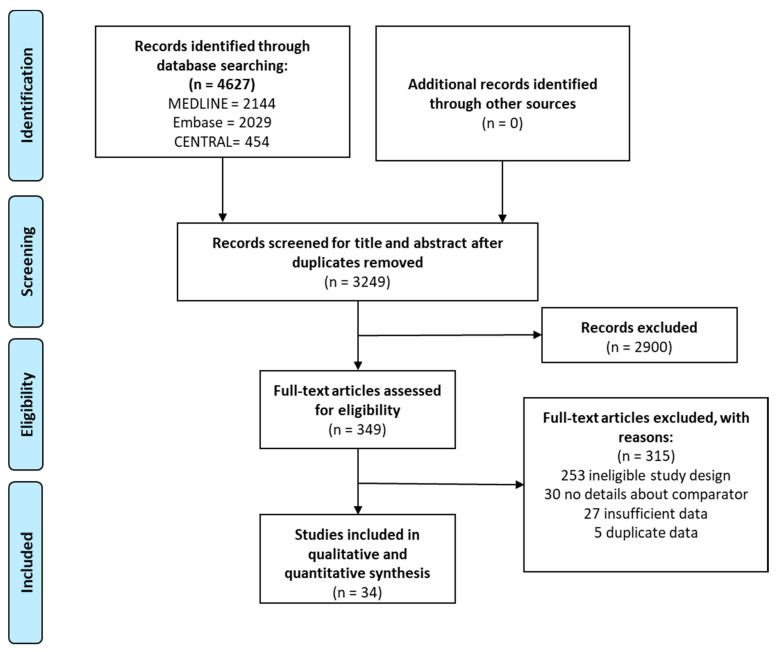
PRISMA flowchart showing the process of systematic search and article selection.

Our search strategy yielded 4627 studies from the three databases. After duplicate removal, we screened 3249 articles by title, abstract and full text, out of which 34 [12,21,22,23,24,25,26,27,28,29,30,31,32,33,34,35,36,37,38,39,40,41,42,43,44,45,46,47,48,49,50,51,52,53,54] were eligible for meta-analysis. No additional reports were identified in the primary eligible studies’ reference lists. The search and selection process are presented in Figure 1.

### 3.2. Characteristics of the Studies Included

The basic characteristics of the included articles are summarized in Table 1. Out of the 34 included articles, 33 were from China [12,21,22,23,24,25,26,27,28,29,31,32,33,34,35,36,37,38,39,40,41,42,43,44,45,46,47,48,49,50,51,52,53,54,55] and 1 from Italy [30]. All of the included studies were conducted during the first wave of the COVID-19 pandemic. Three of them were prospective cohort studies; the rest were retrospective analyses. The proportion of patients with RSP ranged from 2.38% to 58.63%. The follow-up period of patients ranged from 14 to 58 days in the included studies. Data about the PCR and antigen detection techniques are in the Supplementary Material (Tables S1 and S2).

### 3.3. Quantitative Synthesis

Our meta-analysis included 34 studies with 9269 patients reporting on 1490 repeatedly positive SARS-CoV-2 patients; the mean rate of the repositive cases was 16.08%.

#### 3.3.1. Immunoglobulins

**Figure 2:**
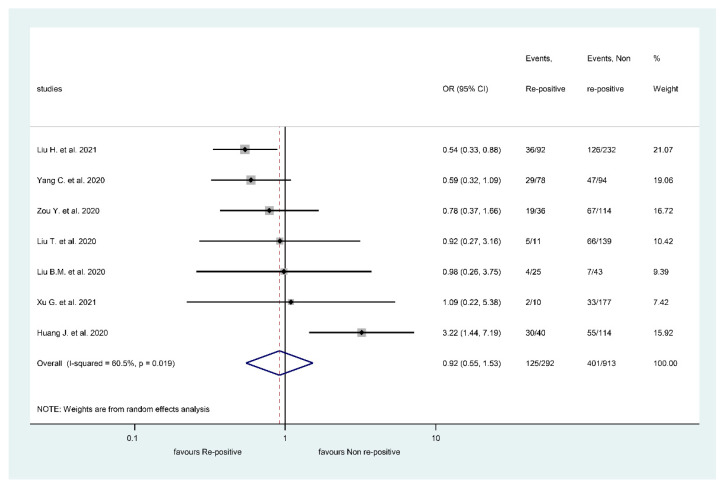
Forest plot representing that repeatedly SARS-CoV-2 positive episode does not significantly impact Immunoglobulin M’s presence.

Based on five articles, IgM positivity did not differ between the RSP positive and RSP negative groups (Figure 2) (OR: 0.92, CI: 0.55, 1.53, *p* = 0.737), IgM levels were positive in 125/292 RSP patients (42.81%) and 401/913 non-RSP patients (43.92%). Heterogeneity for IgM was substantial (I^2^: 60.5, *p* = 0.019) (Figure 2).

**Figure 3:**
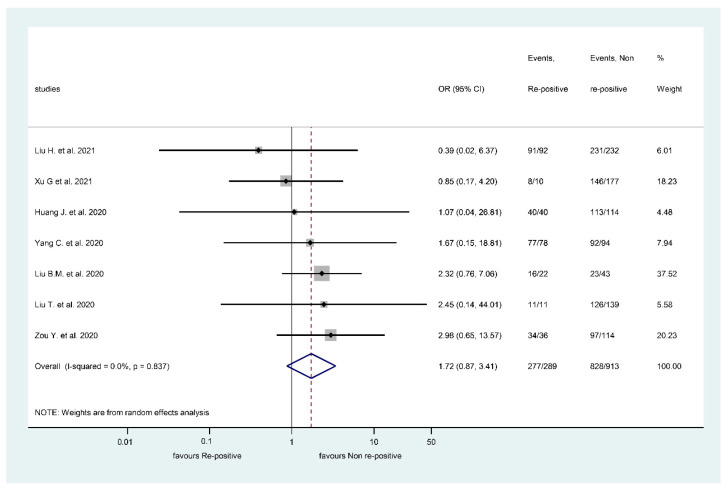
Forest plot representing that repeatedly SARS-CoV-2 positive episode is not associated with Immunoglobulin G positivity.

However, IgG positivity was associated with a higher RSP rate, but the difference was not statistically significant (Figure 3) (OR: 1.72, CI: 0.87, 3.41, *p* = 0.117). IgG levels were positive in 277/289 RSP patients (95.85%) and 828/913 non-RSP patients (90.69%). Heterogeneity was not present in the case of IgG comparison (I^2:^ 0% and *p* = 0.837) (Figure 3).

#### 3.3.2. Risk Factors for Repeated SARS-CoV-2 Positivity

When comparing patients with mild COVID-19 with those with non-mild cases during the first episode, we found decreased odds of presenting repeated PCR positivity in the mild group (Table 2). Except for this comparison, the other risk factors showed no statistically significant difference. 

Forest plots for risk factors are presented in Supplementary Figures S1–S6.

### 3.4. Risk of Bias Assessment

Supplementary Figures S11–S18 show the results of the risk of bias assessment. The publication bias could only be assessed for age, gender, length of hospitalization, and severity of COVID-19 infection. Only when comparing mean differences in age between groups did there appear a significant publication bias (*p* = 0.001).

## 4. Discussion

One of our most important findings is that repeated or prolonged COVID-19-PCR positivity may lead to a higher chance of the presence of IgG antibodies. It might be that those individuals who have long-term viral shedding will have a more prominent immune response. We found that a milder disease course might be associated with a higher rate of repositivity. The underlying cause of this finding could be that the milder infection does not initiate a strong immune response.

A Chinese study of 284 patients reported 100% IgG positivity after recovery from the first infection [11]. An Italian study also described that spike (receptor binding domain) RBD-IgG is present in more than 96.5% of the patients after four weeks of the infection [56]. Although these results look promising, during COVID-19 infection, multiple types of antibodies can develop [11]. A study showed that the antigens’ persistence also depends on the analytical kit used for the detection [57]. These can be associated with different immunity and risk for future PCR positivity, which should be the focus of future investigations. Another issue that can be addressed in more detail in the future is the measurement of neutralizing antibodies, although an increasing number of studies are dealing with the topic. A longitudinal study that involved 517 patients concluded that the dynamic of the neutralizing antibody response in patients who have recovered from COVID-19 infection is variable and could only be assessed on an individual level [58]. Prediction of reinfection or repositivity could be facilitated by knowing the titer of neutralizing antibodies.

Our analysis did not find an association between age and repeated positivity. In a meta-analysis of thirteen studies, the authors describe an increased risk for severe disease course if the patient is older than 65 years [59]. This result indirectly suggests that once elderly patients recover, they are not at risk for repeated positivity, although more data are needed to clarify this question. A population-based study observed that patients younger than 65 are least protected against a repeated infection [14]. We did not find a significantly different gender ratio between the RSP and non-RSP patients.

Several previous works identified obesity as a risk factor for severe COVID-19 infection [60,61]. Despite these findings, we could not prove that BMI is associated with RSP.

Neither the length of hospitalization nor the severity of the first episode seem to influence the risk for RSP. On the other hand, the higher mortality rate of severe and critical disease groups might be a confounding factor. Therefore, our results should be assessed critically, as no previous works attempted to evaluate the potential role of length of hospitalization and severity on repeated positivity.

### 4.1. Strengths and Limitation

Our study group implemented a rigorous methodology while completing the most up-to-date comprehensive work on the topic of potential risk factors of repeated COVID-19 positivity.

This meta-analysis carries multiple limitations. Repeated PCR positivity might be caused by relapse, reinfection, prolonged viral shedding, or false-positive PCR tests. To help with differentiation, we collected available data about viral loads and cyclic threshold (Ct) values that the studies used for the diagnosis. A total of 13 studies reported the Ct values for the PCR positivity, which changed between 37 and 40, which is higher than the recommendation of the Centers for Disease Control and Prevention, which propose a Ct value of 33 [62]. Most of the included articles did not differentiate between these conditions. Furthermore, the follow-up time of the included patients was 14–58 days, which makes re-infection unlikely.

High and moderate risk of bias among the included cohort studies primarily resulted from the differences in patients’ baseline characteristics and the imprecisely defined confounding factors.

### 4.2. Implication for Practice

We suggest the follow-up of COVID-19 convalescent patients in terms of antibody positivity to identify IgG negative patients, as they might be more prone to repeated positivity. We also encourage checking IgG positivity in the general population to assess possible asymptomatic patients’ immune status.

### 4.3. Implication for Research

We emphasize the importance of future follow-up studies on convalescent COVID-19 patients to assess the potential differences and risk factors of repeatedly and non-repeatedly positive patients. We also propose conducting serological studies to have a clearer view of the different immunoglobulins’ roles.

## 5. Conclusions

Our analysis concluded that immunoglobulin G negativity might be associated with repeated COVID-19 PCR positivity. Age, male gender, body mass index, length of hospitalization and severe infection during the first episode do not seem to influence the risk of repeated positivity, although mild initial infection showed higher odds for repeated positivity after hospital discharge or recovery. However, prospective follow-up studies focusing on this topic are required.

## Figures and Tables

**Table 1:** Basic characteristics of the included studies.

Study	Study Design	Follow-Up (Days)	Number of Patients (% Female)	Age (Years) (^a^)	Reported Characteristics	Number of RSP Reported	Rate of RSP (%)	Actual Viral Load/Ct of the RSP Group (^a^)	Ct Value for Diagnosis
An J. et al., 2020 [23]	R	28	262 (48.09)	n.r.	severity	38	14.5	n.r.	≤37.0
Ao Z. et al., 2021 [44]	R	9	51 (39.2)	47 (43–55)	age, gender, LOH, severity	25	49.02 ^c^	n.r.	n.r.
Chen L.Z. et al., 2020 [24]	R	14	76 (57.9)	n.r.	age, gender, LOH, BMI	44	57.89 ^c^	n.r.	n.r.
Chen S. L. et al., 2020 [25]	R	28	1282 (51.17)	43 (32–57)	age, gender, severity, LOH	189	14.74	n.r.	n.r.
Du H. et al., 2020 [26]	R	14	126 (51.6)	66 (54–69)	age, gender, severity, LOH	3	2.38	n.r.	≤37
Hao Y et al., 2020 [43]	R	30	369 (49.6)	n.r.	gender	23	6.23	n.r.	n.r.
He S. et al., 2020 [12]	R	14	267 (57)	57 (37–68)	age, gender, severity, LOH	30	11.23	n.r.	n.r.
Hong L. et al., 2021 [45]	P	48	145 (46.9)	47 (10–86)	age, gender, LOH, BMI, severity	13	8.97	n.r.	≤40
Hu F. et al., 2020 [27]	R	n.r.	178 (53.93)	46.74 (2–90)	age, LOH	20	11.23	30 (24–40)	≤40
Hu J. et al., 2020 [28]	P	n.r.	117 (58.1)	49.32 ± 11.93	age, gender,	8	6.38	30.91 ±2.50	n.r.
Hu R. et al., 2020 [21]	R	14	69 (49.3)	33 (2–78)	gender, severity	11	15.94	n.r.	n.r.
Huang J. et al., 2020 [29]	R	28	414 (47)	n.r.	gender, antibodies, severity, BMI	69	16.67	32 (29–37)	≤37
Landi F. et al., 2020 ^b^ [30]	P	55.8 ± 20.8	131 (38.9)	55.8 ± 14.8	age, gender, LOH, BMI	22	16.79	n.r.	n.r.
Liu B. M. et al., 2020 [31]	R	13.8 ± 6.1	68 (63.2)	44.3 ± 16	gender, antibodies	25	36.76	n.r.	n.r.
Liu C. et al., 2020 [32]	R	14	51 (58.2)	46.6 ± 13.9	LOH	9	17.64	n.r.	n.r.
Liu H. et al., 2021 [46]	R	n.r.	354 (52.2)	n.r.	age, gender, LOH, antibodies, severity	92	28.39	n.r.	≤37
Liu J. et al., 2021 [47]	R	n.r.	111 (36.9)	n.r.	age, gender, severity	47	42.34	n.r.	≤38
Liu T. et al., 2020 [33]	R	n.r.	150 (50.67)	n.r.	age, gender, antibodies	11	7.34	n.r.	n.r.
Liu Y. et al., 2021 [48]	R	4–20	90 (45.6)	49 (33–60)	age, gender	10	11	n.r.	n.r.
Lu J. et al., 2020 [34]	R	14	619 (n.r.)	n.r.	age, gender, LOH	87	14.05	n.r.	n.r.
Shi L. et al., 2021 [49]	R	n.r.	98 (43.9)	57.5 (15–83)	gender	15	15.3	n.r.	n.r.
Shui T.J. et al., 2020 [35]	R	n.r.	758 (48.6)	46.1 ± 16.82	age, gender, severity	59	7.78	n.r.	≤37
Xiao J. et al., 2020 [36]	R	n.r.	116 (75)	45.32	age, gender,	40	34.48	n.r.	n.r.
Xu G. et al., 2021 [50]	R	45.7	187 (56.15)	n.r.	antibodies	10	5.35	n.r.	n.r.
Yan N. et al., 2020 [37]	R	14	337 (54.3)	44 (34–55)	age, gender, severity, LOH	25	7.42	n.r.	n.r.
Yang C. et al. 2020 [38]	R	14	479 (53.2)	n.r.	age, gender, antibodies, severity, LOH	93	19.51	35 (95% CI: 35–36)	≤40
Yang Z. et al., 2021 [51]	R	14	79 (35)	n.r.	age, gender, LOH, severity	7	9.4	n.r.	n.r.
Ye H. et al., 2020 [39]	R	14	117 (44.4)	48.2 ± 13.5	age, gender, LOH	12	10.25	n.r.	n.r.
Yuan B. et al., 2020 [22]	R	14	182 (53.8)	46.4 ± 17.1	severity, LOH	20	10.98	n.r.	≤40
Zhang J. et al., 2021 [52]	R	180	527 (23.3)	42.5 (32–54)	age, gender, LOH, severity	32	6.07	n.r.	n.r.
Zhao H. et al., 2021 [53]	R	n.r.	411	202 (n.r.)	age, gender, BMI, severity	241	58.63	n.r.	n.r.
Zheng J. et al., 2020 [40]	P	15	285 (55.1)	48 (35–62)	age, gender, severity, LOH	27	9.47	35 (34–39)	≤37
Zhou J. et al., 2020 [54]	R	n.r.	368 (50)	51 (40–62)	age, gender, LOH, severity	23	6.67	n.r.	≤38
Zhu H. et al., 2020 [41]	R	28	98 (67.3)	52 (37.8–59)	age, gender, LOH, BMI	17	17.34	n.r.	≤40
Zou Y. et al., 2020 [42]	R	n.r.	257 (47.85)	n.r	gender, antibodies, severity	53	20.62	n.r.	n.r.

All studies were published during the year 2020; ^a^ as reported by: mean ± SD, median (IQR/range); min = minimum, max = maximum; ^b^ this study is from Italy, while all the others are from China; ^c^ case-control studies, abbreviations: n.r. = not reported, R = retrospective; P = prospective; RSP = repeated SARS-CoV-2 positivity, LOH = length of hospital stay; BMI = body mass index.; Ct = Cyclic threshold.

**Table 2:** Results of the analyses on risk factors.

**Risk Factor**	**Effect Size in OR**	**95% CI**	***p*-Value**	**Heterogeneity (I^2^) (in%)**	***p*-Value for the χ^2^ Test of Heterogeneity**
**Mild first episode**	**0.3**	**0.14–0.67**	**0.003**	**81**	**<0.001**
Severe first episode	0.66	0.41–1.07	0.095	62.6	<0.001
Gender	1.10	0.97–1.25	0.146	0	0.57
**Risk Factor**	**Effect Size in WMD**	**95% CI**	***p*-Value**	**Heterogeneity (I^2^) (in%)**	***p*-Value for the χ^2^ Test of Heterogeneity**
Age	−1.93 years	−4.49–0.63	0.139	84.1	<0.001
Body mass index	0.10 kg/m^2^	−0.72–0.92	0.818	60.5	0.027
Length of hospital stay	−0.14 days	−1.50–1.22	0.837	81.9	<0.001

**Bold text:** Statistically significant difference.

## Data Availability

The datasets used in this study can be found in the full-text articles used in this systematic review.

## Supplementary Materials

https://www.mdpi.com/article/10.3390/v13050809/s1, Figure S1: Forest plot representing that patients’ age does not have a significant impact on repeated SARS-CoV-2 positivity, Figure S2: Forest plot representing that patients’ gender does not have a significant impact on repeated SARS-CoV-2 positivity, Figure S3: Forest plot representing that body mass index does not have a significant impact on repeated SARS-CoV-2 positivity, Figure S4: Forest plot representing that the severity of the initial infection does have a significant impact on repeated SARS-CoV-2 positivity, Figure S5: Forest plot representing that severity of the initial infection does not have a significant impact on repeated SARS-CoV-2 positivity, Figure S6: Forest plot representing that length of hospital stay during the initial infection does not have a significant impact on repeated SARS-CoV-2 positivity, Figure S7: Funnel plot of the studies reporting on age of the patient, Figure S8: Funnel plot of the studies reporting on sex of the patient, Figure S9: Funnel plot of the studies reporting on the rate of severe cases during the initial infection, Figure S10: Funnel plot of the studies reporting on the length of hospital stay during the initial infection, Figure S11: Risk of bias assessment on study level [A] and across studies [B] comparing patients age in the repeatedly SARS-CoV-2 positive and non-positive patients, Figure S12: Risk of bias assessment on study level [A] and across studies [B] comparing patients gender in the repeatedly SARS-CoV-2 positive and non-positive patients, Figure S13: Risk of bias assessment on study level [A] and across studies [B] comparing IgG positivity in the repeatedly SARS-CoV-2 positive and non-positive patients, Figure S14: Risk of bias assessment on study level [A] and across studies [B] comparing IgM positivity in the repeatedly SARS-CoV-2 positive and non-positive patients, Figure S15: Risk of bias assessment on study level [A] and across studies [B] comparing the proportion of severe cases in the first infection in repeatedly SARS-CoV-2 positive and non-positive patients, Figure S16: Risk of bias assessment on study level [A] and across studies [B] comparing the proportion of mild cases in the first infection in repeatedly SARS-CoV-2 positive and non-positive patients, Figure S17: Risk of bias assessment on study level [A] and across studies [B] comparing length of hospital stay during the first infection in repeatedly SARS-CoV-2 positive and non-positive patients, Figure S18: Risk of bias assessment on study level [A] and across studies [B] comparing body mass index (BMI) in repeatedly SARS-CoV-2 positive and non-positive patients.
