# Supplementary material for: Immunoglobulin Response and Prognostic Factors in Repeated SARS-CoV-2 Positive Patients: A Systematic Review and Meta-Analysis
Source: Viruses. 2021 Apr 30;13(5):809. doi: 10.3390/v13050809 (PMC8146492; doi:10.3390/v13050809)
Supplement: Supplementary file 1 [file viruses-13-00809-s001.zip › viruses-1166490-supplementary.pdf]

Study	RT-PCR techniques used in the studies
An J. *et al.2020 [26]*	Total nucleic acid extraction from the samples was performed using the QIAamp RNA Viral Kit (Qiagen, Heiden, Germany). Quantitative RT-PCR was undertaken using a China Food and Drug Administration (CFDA) approved commercial kit specific for 2019-nCoV detection (GeneoDX Co., Ltd., Shanghai, China) or Sherlock kit. Spike genes and ORF1 genes were detected using this hyper-sensitive method.
Ao Z. *et al. 2021[47]*	RT-PCR test kit (Coyotebio, Beijing, China) recommend by the Chinese Center for Disease Control and Prevention (CCDC).
Chen L.Z. *et al. 2020 [27]*	Not reported
Chen S. L. *et al. 2020 [28]*	Total RNA was extracted using a prefilled viral total NA kit-Flex (catalog no. KFRPF-805296, Labserv; Fisher Scientific, https://www.fishersci.com) following manufacturer's instructions. A commercial RT-PCR assay kit targeting the ORF1ab and N genes was used to detect SARS-CoV-2 RNA (catalog no. DA0931; DaAn Gene, Guangzhou, China). Amplification was performed on an Applied Biosystems 7500 Real-Time PCR System (Thermo Fisher Scientific, https:// www.thermofisher.com) as follows: 50°C for 15 minutes and then 95°C for 15 minutes, followed by 45 cycles of 94°C for 15 seconds and 55°C for 45 seconds. When both ORF1ab and N gene target amplification curves were generated within 40 cycles, the samples were considered to be positive for SARSCoV-2 RNA.
Du H. *et al. 2020 [29]*	
Hao Y *et al. 2020[46]*	real-time reverse-transcription polymerase chain reaction (RT-PCR) kit (Bio-Germ, Shanghai, China) targeting the open reading frame 1ab (ORF1ab) gene and nucleocapsid protein (N) gene, recommended by the Chinese Center for Disease Control and Prevention
He S. *et al. 2020 [15]*	Not reported
Hong L. *et al. 2021 [48]*	The RNA detection kits were provided by Sansure Biotech (Changsha, China) and ZJ Bio-Tech (Shanghai, China), and used according manufacturer's protocol by specialized laboratory personnel.
Hu F. *et al. 2020 [30]*	The real-time reverse transcriptase-polymerase chain reaction (RT-PCR) kit (Wuhan Easydiagnosis Biomedicine Co. Ltd
Hu J. *et al. 2020 [31]*	Viral RNA was extracted using a Nucleic Acid Isolation Kit (Da'an Gene Corporation, Cat: DA0630) on an automatic workstation Smart 32 (Da'an Gene Corporation) according to the instructions. Real-time reverse transcription polymerase chain reaction (RT-PCR) reagent (Da'an Gene cooperation, Cat: DA0930), which targets the N and orf1ab genes, was employed for viral detection per the protocol
Hu R. *et al. 2020 [24]*	
Huang J. *et al. 2020 [32]*	
Landi F. *et al. 2020a[33]*	Fluorescence quantitative polymerase chain reaction; Applied Biosystems
Liu B. M. *et al. 2020 [34]*	
	Not reported
Liu C. *et al. 2020 [35]*	Total RNA was extracted from the clinical specimens using the QIAamp RNA Viral Kit (Qiagen, Heiden, Germany). A qRT-PCR Test Kit (product code. GZ-D2RM25, Shanghai GeneoDx Biotech Co., Ltd) targeting the ORF1ab and N genes of SARS-CoV-2 was used. A cycle threshold (Ct)value less than 37 was interpreted as positive for SARS-CoV-2 RNA1
Liu H. *et al. 2021[49]*	
Liu J. et al. 2021 [50]	Not reported
Liu T. *et al. 2020[36]*	Not reported
Liu Y. *et al. 2021[51]*	
	The oropharyngeal swab was tested using the Coronavirus Disease 2019 (ORF lab/N gene) nucleic acid detection kit (Shanghai BioGerm Medical Biotechnology Co., Ltd). The detection kit was officially approved by the National Medical Products Administration and received European Union CE certification.
	Not reported
Lu J. *et al. 2020 [37]*	Not reported
	Not reported
	Not reported
Shi L. *et al. 2021[52]*	
Shui T.J. *et al. 2020 [38]*	For RT-PCR diagnosis, total RNA was extracted from clinical specimens using the QIAamp Viral RNA mini kit (QIAGEN, Germany) according to the manufacturer's instructions. In this study, three RT-PCR kits were used to conduct nucleic acid testing, in an attempt to avoid false negatives. Kit A (DAAN GENE, Guangzhou, China)and Kit B (BioGerm, Shanghai, China) have primers and probes targeting the open reading frame (ORF1ab) and nucleocapsid protein (N), respectively. Kit C (Liferiver, Shanghai, China) is designed to detect RNA-dependent RNA polymerase (RdRp), envelope protein (E) and N. Kit A and Kit C were included into WHO Emergency Use Listing for detecting SARS-CoV-2 nucleic acid (https://www.who.int/diagnostics_laboratory/200710_eul_sars_cov2_product_list.pdf?ua=1).
Xiao J. *et al. 2020 [39]*	
Xu G. *et al.* 2021 [53]	
Yan N. *et al. 2020 [40]*	
Yang C. *et al. 2020 [41]*	Not reported
Yang Z. *et al. 2021[54]*	
Ye H. *et al.* 2020 [42]	Real-time RT-PCR was performed on throat swab specimens at Wuhan Ping an Hao Medical Laboratory according to the protocol from DAAN Gene Co., Ltd., of Sun Yat-sen University. SARS-CoV-2 open reading frame 1ab (ORF1ab) and nucleocapsid protein (NP) gene fragments were amplified, and the conditions for amplification were 50 °C for 10 min and 97 °C for 1 min, followed by 40 cycles of 97 °C for 5 s and 58 °C for 30 s. When both targets (ORF1ab and NP) tested positive by specific real-time RT-PCR, the case was considered to be laboratory-confirmed.
	SARS-CoV-2 was detected by RT-PCR assay using a COVID-19 Nucleic Acid Detection Kit according to the manufacturer's protocol (Shanghai Huirui Biotechnology Co, Ltd).
Yuan B. *et al. 2020 [25]*	Not reported<br>Not reported
	SARS-CoV-2 RT-qPCR tests were performed on the day of sampling using commercial kits (Zhongshan Daan Biotech).
	Not reported
Zhang J. *et al. 2021[55]*	
Zhao H. *et al. 2021[56]*	RT-PCR detection reagents were provided by Shanghai BioGerm Medical Biotechnology Co., Ltd. The primers sequences were as follows: forward primer 50-CCCTGTGGGTTTTACACTTAA-30; reverse primer 50- ACGATTGTGCATCAGCTGA- 30 . Conditions for the amplifications were 50 °C for 15 min, 95 °C for 3 min, followed by 45 cycles of 95 °C for 15 s and 60 °C for 30 s.
Zheng J. *et al. 2020 [43]*	
Zhou J. *et al. 2020[57]*	
Zhu H. *et al. 2020 [44]*	The RT-PCR tests were performed by the CDC using the High Pure Viral RNA Kit (Roche, Mannheim, Germany) and the 2019-nCoV Viral RNA detection kit (Bio-Germ, Shanghai, China). Two target genes of SARS-CoV-2, including the open reading frame 1ab (ORF1ab) and the nucleocapsid protein (N), were simultaneously amplified and tested during the RT-PCR assay. Target 1 (ORF1ab): forward primer CCCTGTGGGTTTTACACTTAA; reverse primer ACGATTGTGCATCAGCTGA; probe: 5′-VIC-CCGTCTGCGGTATGTGGAAAGGTTATGG-BHQ1-3′. Target 2 (N): forward primer GGGGAACTTCTCCTGCTAGAAT; reverse primer CAGACATTTTGCTCTCAAGCTG; probe 5′-FAM- TTGCTGCTGCTTGACAGATT-TAMRA-3′.
	Pathogen testing was performed using an ORF1ab/N Gene Nucleic acid detection kit (BioGerm, China), following the manufacturer's instructions. The assay targeted the SARS-CoV-2N gene and the ORF1ab gene.
Zou Y. *et al. 2020[45]*	Not reported
	Not reported

The samples were subsequently tested using quantitative reverse-transcription PCR to detect SARS-CoV-2 RNA. The open reading frame 1ab (ORF1ab) and nucleocapsid protein (N) were the two target genes. Total nucleic acid was extracted within 2 h using the respiratory sample RNA isolation kit (Shanghai BioGerm Medical Biotechnology CoLtd) according to the manufacturer's instructions. The sequences for the ORF1ab real time RT-PCR were as follows: forward primer: CCCTGTGGGTTTTACACTTAA; reverse primer: ACGATTGTGCATCAGCTGA; probe: 5'-FAM-CCGTCTGCGGTATGTGGAAAGGTTATGG-BHQ1-3'. The sequences for the N gene were as follows: forward primer: GGGGAACTTCTCCTGCTAGAAT; reverse primer: CAGACATTTTGCTCTCAAGCTG; probe:5'-FAM-TTGCTGCTGCTTGACAGATT-TAMRA-3.

Not reported

RT-PCR tests were performed on throat swabs using 2019-nCoV test kits (Wondfo, China). The molecular technique mainly focused on the detection of the open reading frame lab (ORFlab) and nucleocapsid protein (N) regions of the SARS-CoV-2 genome, and the CT value was determined according to the manufacturer's instructions. Additionally, any suspicious results resulted in retesting.

Table S1 PCR techniques used in the certain studies

Study name	Serological assey/kit used for the antibody detection
Huang J. et al	SARS-CoV-2 antibody Chemiluminescent microparticle immunoassay (CMIA) kit (Innodx, Xiamen, China; catalog no. Gxzz 20203400198). Total antibody detection was based on double-antigens sandwich methodology and the IgM antibody detection was based on μ-chain capture immunoassay.
Liu B.M. et al	not reported
Liu T. et al	COVID-19 IgM/IgG chemiluminescence test kit on a fully-automated chemiluminescence immunoassay analyzer. The test kit contained recombinant SARS-CoV-2 antigen (spike protein and nucleocapsid protein) labelled with magnetic beads, anti-human IgM monoclonal antibody, and anti-human IgG monoclonal antibody
Yang C. et al	Levels of IgM, IgG, and total antibody against the SARS-CoV-2 surface spike protein receptor binding domain in serum specimens were measured by using a chemiluminescence kit (Beijing Wantai Biotech) with a Caris200 automatic chemiluminescence instrument and are represented as cut-off indexes (COIs)
Zou Y. et al	The expression of IgM and IgG antiviral antibodies in serum samples was detected by an automatic chemiluminescence immunoassay technique according to the manufacturer's instructions. This detection system was developed based on the RBD of the recombinant S polypeptide (rS) of SARS-CoV2 and recommended by National Health Commission of China. The specificity and reliability of this kit have been confirmed by Spicuzza and Li [9,10].The analyzer automatically calculated the levels of IgM or IgG (AU/ml) against SARS-CoV-2 according to the RLU and the built-in calibration curve. A result of >10.0 AU/ml was considered positive, while a result of < 10.0 AU/ml was considered negative.
Liu H. et al. 2021	SARS-CoV-2 testing kit (WANTAI BioPharm, Beijing, China) using the chemiluminescence method. All the tests were performed according to the manufacturer's instructions. An S/CO
Xu G et al. 2021	Colloidal Gold-based Immunoassays (Colloidal gold kits, Livzon Inc, Zhuhai, China).

Table S2 Serological techniques used for antibody detection in certain studies

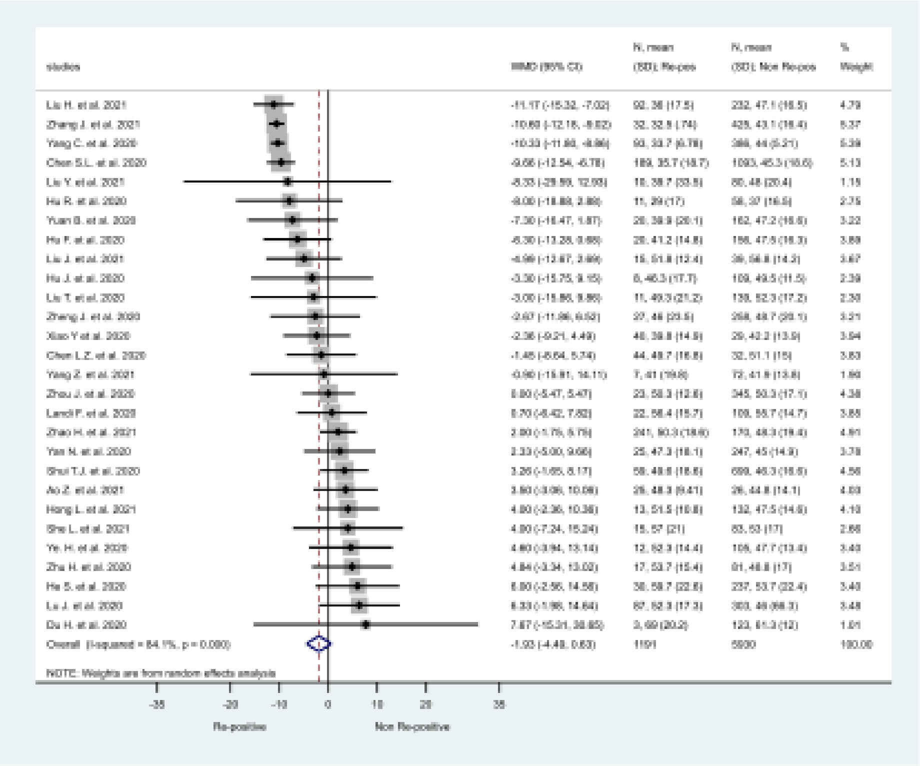

Figure S1: Forest plot representing that patients' age does not have a significant impact on repeated SARS-CoV-2 positivity

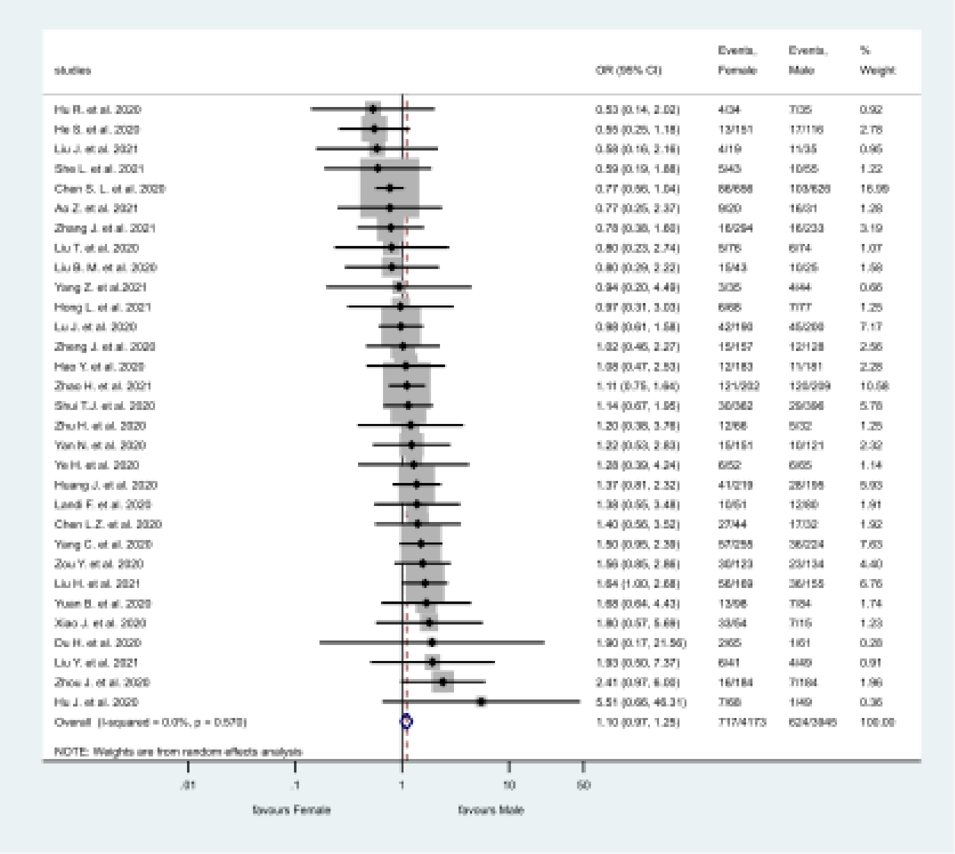

Figure S2 Forest plot representing that patients' gnder does not have a significant impact on repeated SARS-CoV-2 positivity

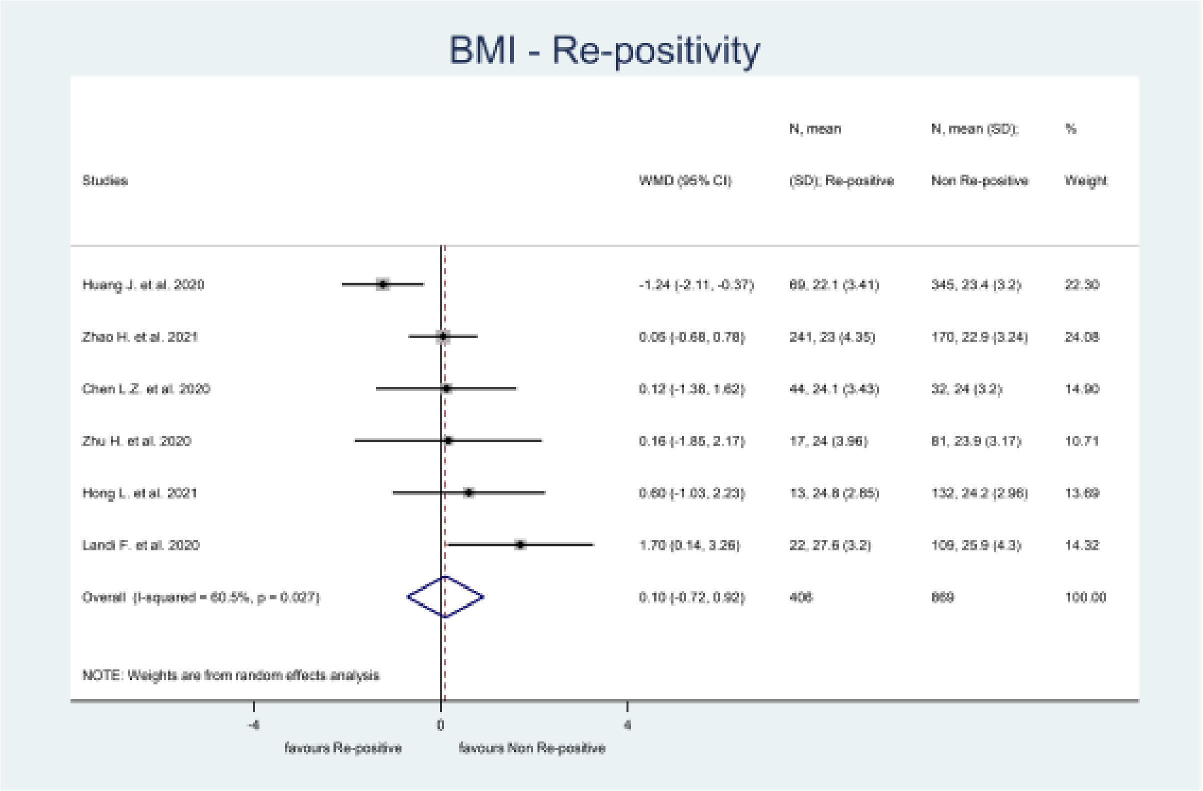

Figure S3: Forest plot representing that body mass index does not have a significant impact on repeated SARS-CoV-2 positivity

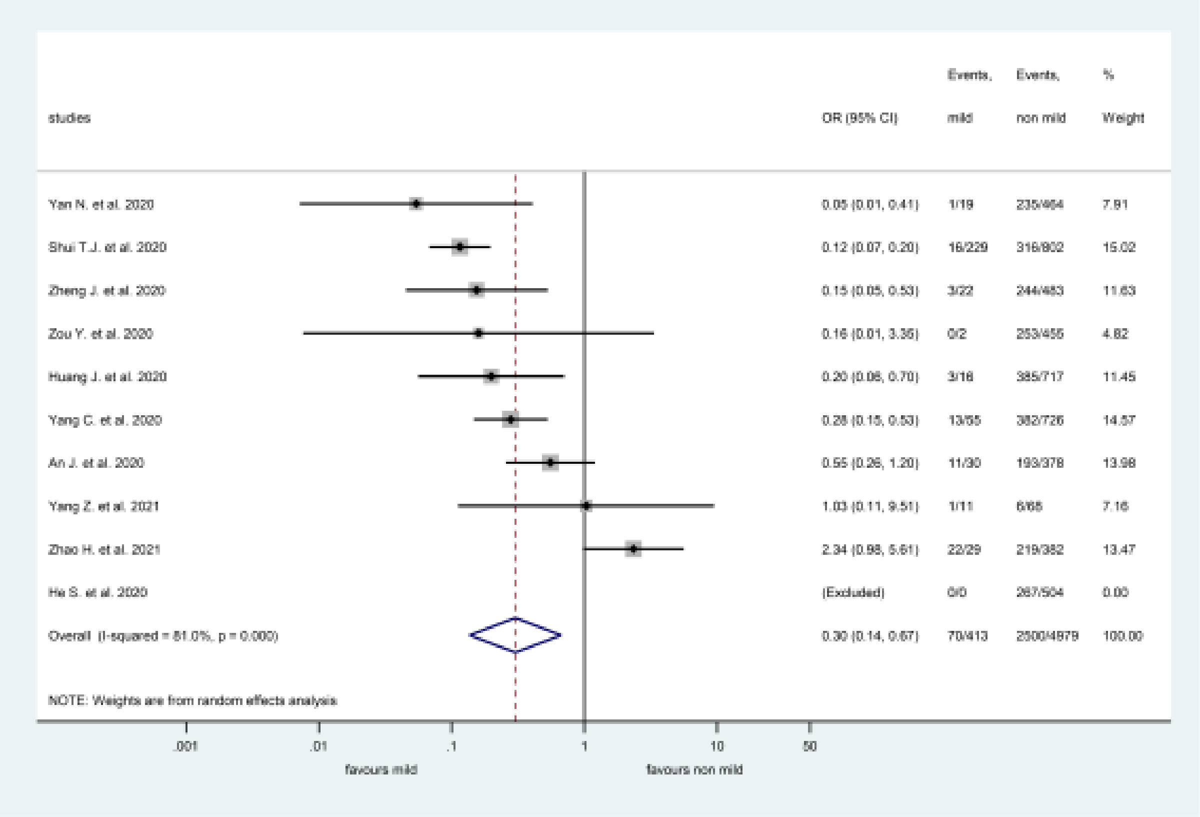

Figure S4 Forest plot representing that severity of the initial infection does have a significant impact on repeated SARS-CoV-2 positivity

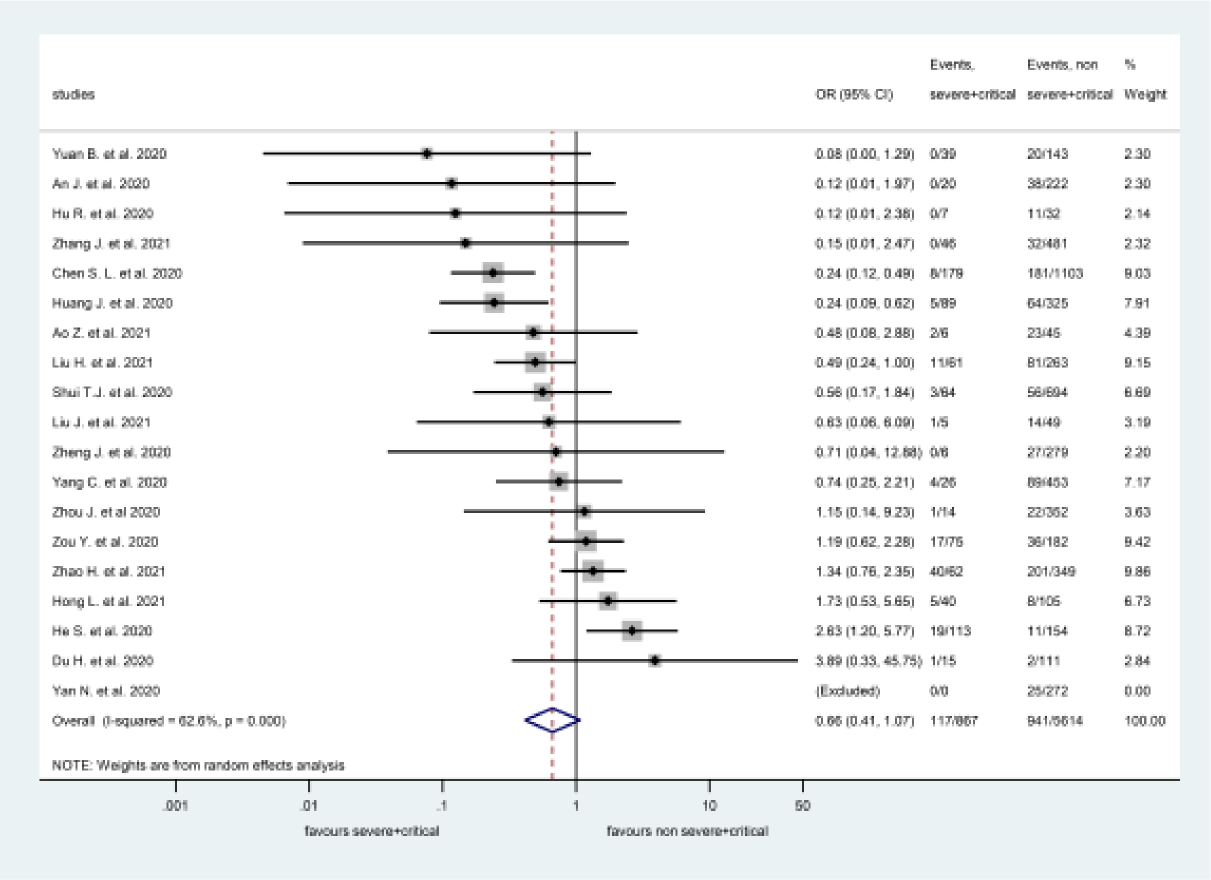

Figure S5: Forest plot representing that severity of the initial infection does not have a significant impact on repeated SARS-CoV-2 positivity

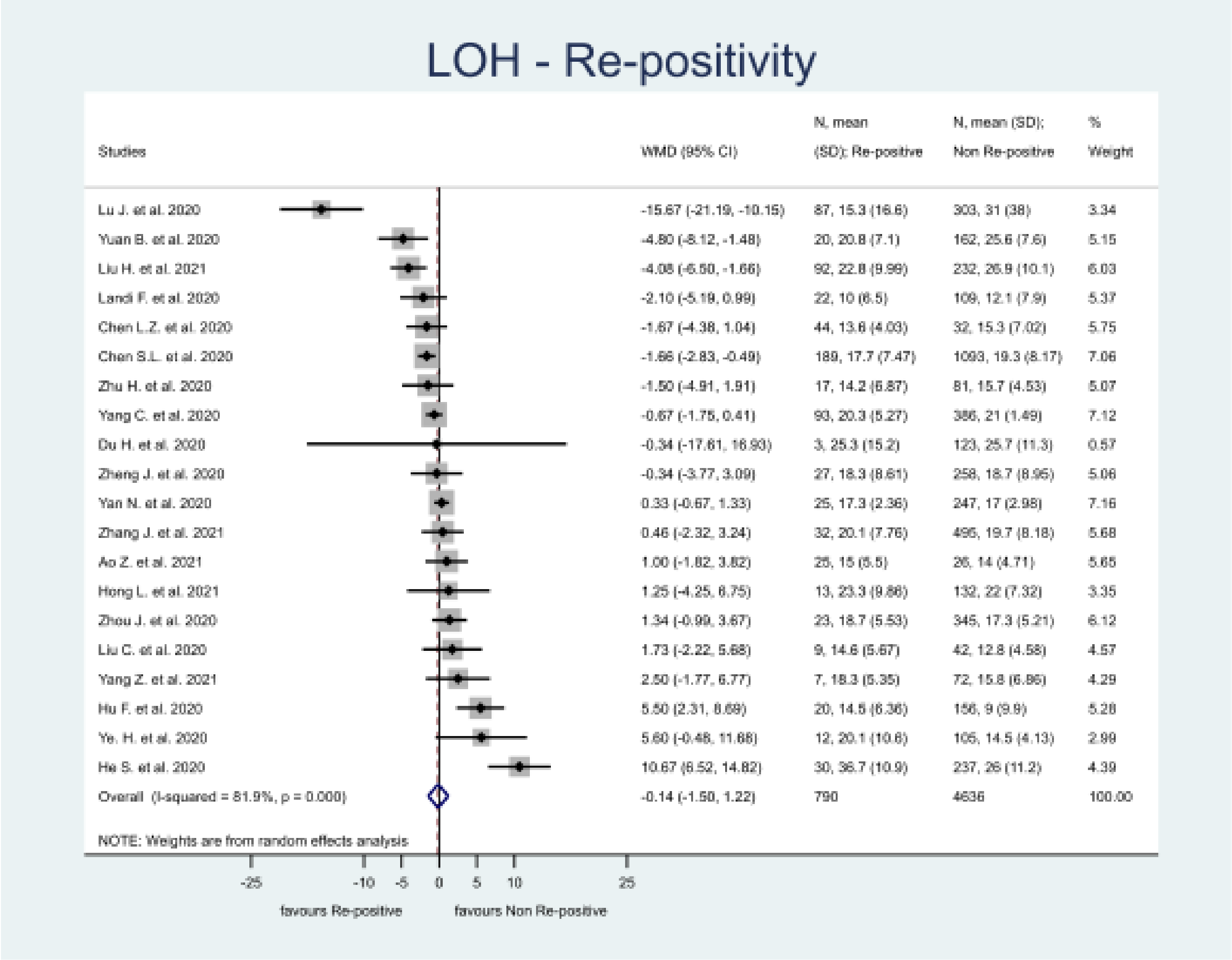

Figure S6: Forest plot representing that length of hospital stay during the initial infection does not have a significant impact on repeated SARS-CoV-2 positivity

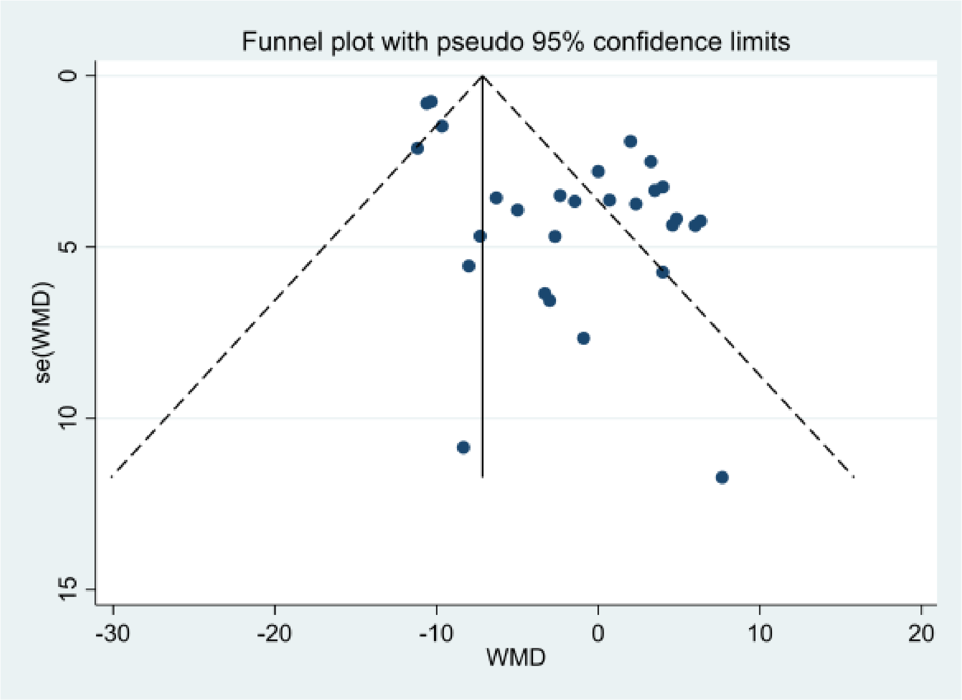

Figure S7: Funnel plot of the studies reporting on age of the patient. The visual assessment of the funnel plot and the Egger's test (p=0.000) did not indicate asymmetry, accordingly small study effect is not likely to be present.

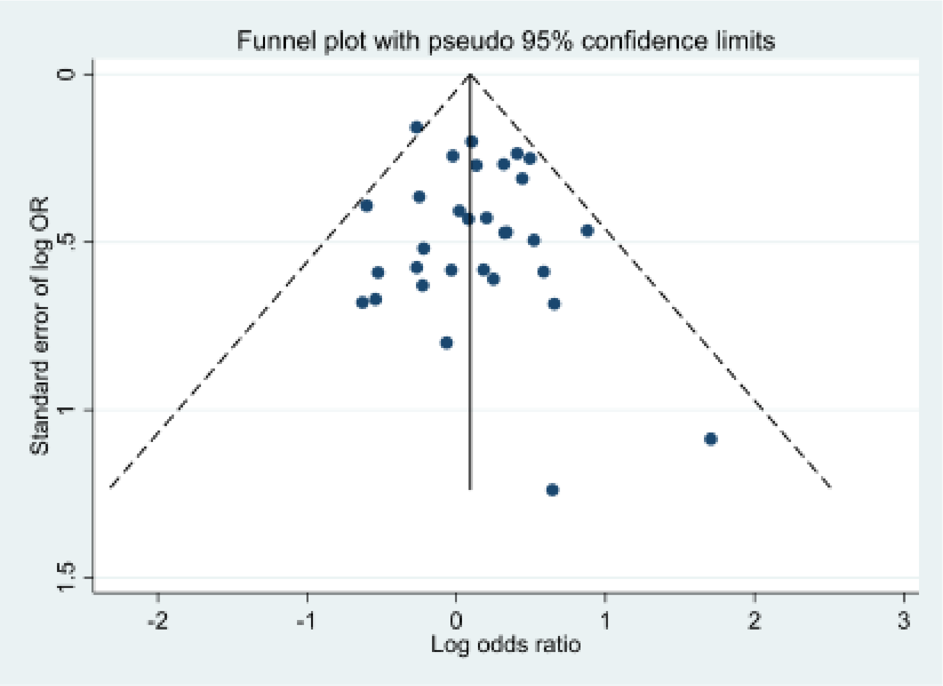

Figure S8: Funnel plot of the studies reporting on sex of the patient. The visual assessment of the funnel plot and the Egger's test (p=0.133) did not indicate asymmetry, accordingly small study effect is not likely to be present.

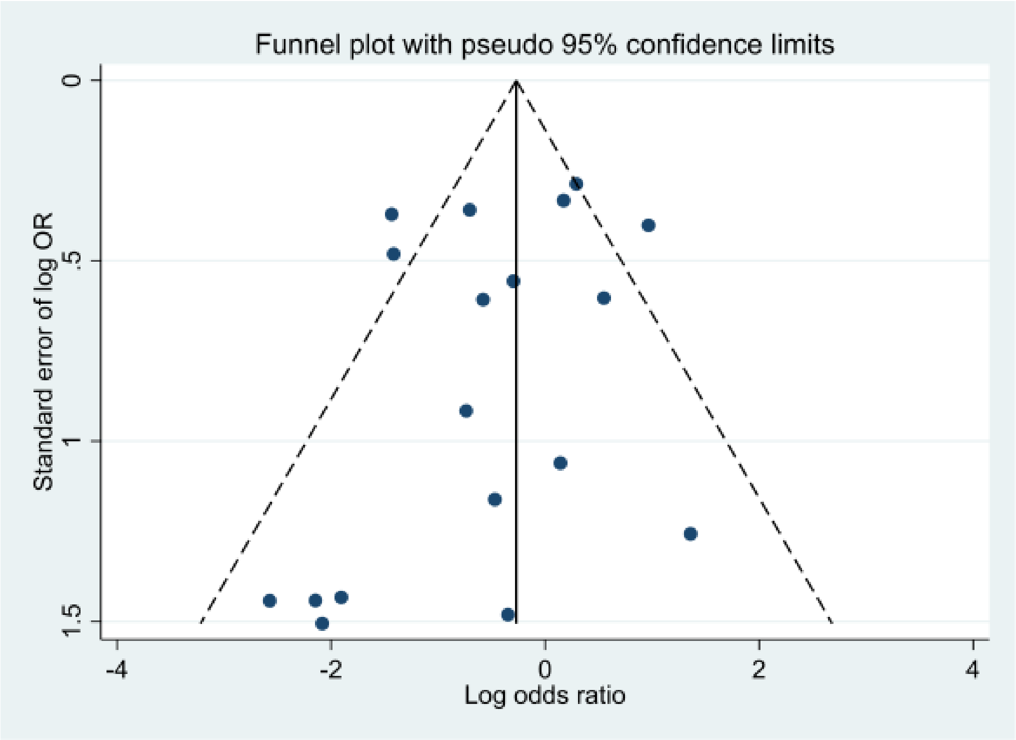

Figure S9: Funnel plot of the studies reporting on the rate of severe cases during the initial infection . The visual assessment of the funnel plot and the Egger's test (p=0. 459) did not indicate asymmetry, accordingly small study effect is not likely to be present.

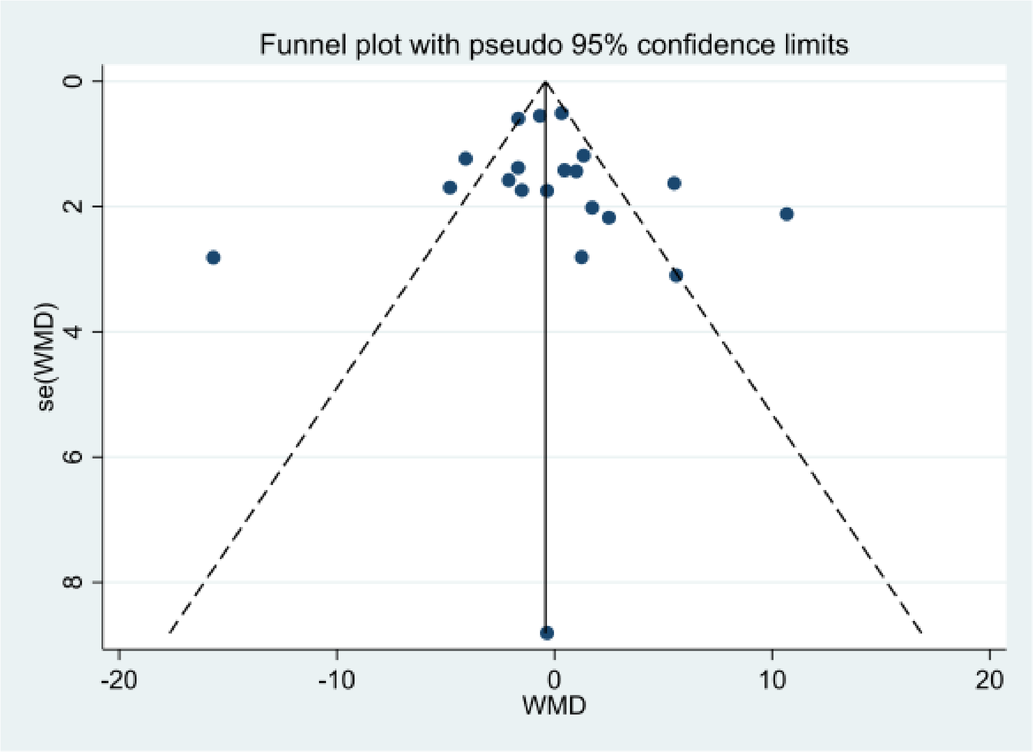

Figure S10: Funnel plot of the studies reporting on the length of hospital stay during the initial infection . The visual assessment of the funnel plot and the Egger's test (p=0. 892) did not indicate asymmetry, accordingly small study effect is not likely to be present.

A

N.a. Not applicable
+ Low risk
? Moderate risk
- High risk

Study	Study participation	Study attrition	Prognostic factor measurement	Outcome measurement	Study confounding	Statistical analysis reporting	Overall risk of bias
Hu R. et al. 2020	+	n.a	+	+	?	+	+
Yuan B. et al. 2020	+	n.a	+	+	-	?	-
Chen L.Z. et al. 2020	+	n.a	+	+	?	+	+
Chen S. L. et al. 2020	+	n.a	+	+	?	+	+
Du H. et al. 2020	+	n.a	+	+	?	+	+
He S. et al. 2020	+	n.a	+	+	+	+	+
Hu F. et al. 2020	?	n.a	?	+	?	+	-
Hu J. et al. 2020	?	?	?	+	?	?	-
Landi F. et al. 2020	+	n.a	+	+	?	+	+
Liu T. et al. 2020	+	n.a	+	+	?	+	+
Lu J. et al. 2020	+	n.a	+	+	?	+	+
Shui T.J. et al. 2020	+	n.a	+	+	?	+	+
Xiao J. et al. 2020	?	n.a	+	+	?	+	?
Yan N. et al. 2020	+	n.a	?	+	?	+	?
Yang C. et al. 2020	+	n.a	+	+	+	+	+
Ye H. et al. 2020	+	n.a	+	+	?	+	+
Zheng J. et al. 2020	?	?	+	+	?	+	-
Zhu, H. et el. 2020	?	n.a	+	+	?	+	?
Zou Y. et al. 2020	?	n.a	+	+	?	+	?
Ao Z. et. al. 2021	+	n.a.	?	+	+	+	+
Hong L. et al. 2021	+	-	+	+	?	+	-
Liu H. et al. 2021	?	n.a.	+	+	?	+	?
Liu J. et al. 2021	?	n.a.	?	+	+	+	?
Liu Y. et al. 2021	+	n.a.	+	+	?	+	+
Shi L. et al. 2021	+	n.a.	?	+	?	+	?
Xu G. et al. 2021	?	n.a.	+	+	+	+	+
Yang Z. et al. 2021	?	n.a.	+	+	?	+	?
Zhou J. et al. 2020	+	n.a.	+	+	?	+	+
Zhang J. et al. 2021	+	n.a.	?	+	?	+	?
Zhao H. et al. 2021	?	n.a.	?	+	?	+	-

B

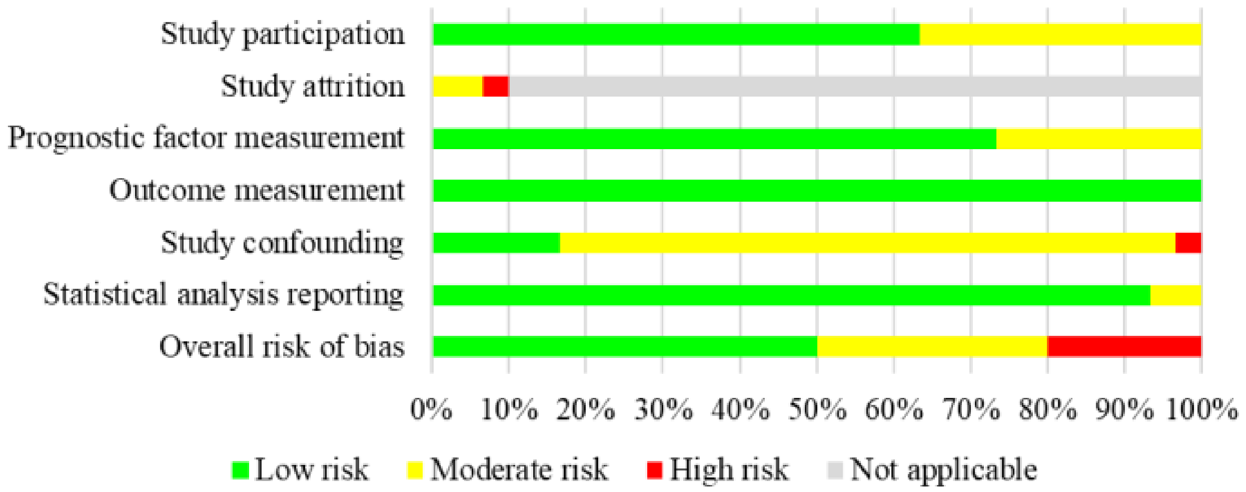

Figure S11: Risk of bias assessment on study level [A] and across studies [B] comparing patients age in the repeatedly SARS-CoV-2 positive and non positive patients.

A

N.a. Not applicable; + Low risk; ? Moderate risk; – High risk

Study	Study participation	Study attrition	Prognostic factor measurement	Outcome measurement	Study confounding	Statistical analysis reporting	Overall risk of bias
Hu R. et al. 2020	+	n.a	+	+	?	+	+
Yuan B. et al. 2020	+	n.a	+	+	-	?	-
Chen L.Z. et al. 2020	+	n.a	+	+	?	+	+
Chen S. L. et al. 2020	+	n.a	+	+	?	+	+
Du H. et al. 2020	+	n.a	+	+	?	+	+
He S. et al. 2020	+	n.a	+	+	+	+	+
Hu F. et al. 2020	?	n.a	?	+	?	+	-
Hu J. et al. 2020	?	?	?	+	?	?	-
Huang J. et al. 2020	+	n.a	+	+	?	+	+
Landi F. et al. 2020	+	n.a	+	+	?	+	+
Liu B. M. et al. 2020	?	n.a	+	+	?	+	+
Liu C. et al. 2020	?	n.a	?	+	?	?	-
Liu T. et al. 2020	+	n.a	+	+	?	+	+
Lu J. et al. 2020	+	n.a	+	+	?	+	+
Shui T.J. et al. 2020	+	n.a	+	+	?	+	+
Xiao J. et al. 2020	?	n.a	+	+	?	+	?
Yan N. et al. 2020	+	n.a	?	+	?	+	?
Yang C. et al. 2020	+	n.a	+	+	+	+	+
Ye H. et al. 2020	+	n.a	+	+	?	+	+
Zheng J. et al. 2020	?	?	+	+	?	+	-
Zhu, H. et el. 2020	?	n.a	+	+	?	+	?
Zou Y. et al. 2020	?	n.a	+	+	?	+	?
Ao Z. et. al. 2021	+	n.a.	?	+	+	+	+
Hong L. et al. 2021	+	-	+	+	?	+	-
Liu H. et al. 2021	?	n.a.	+	+	?	+	?
Liu J. et al. 2021	?	n.a.	?	+	+	+	?
Liu Y. et al. 2021	+	n.a.	+	+	?	+	+
Shi L. et al. 2021	+	n.a.	?	+	?	+	?
Yang Z. et al. 2021	?	n.a.	+	+	?	+	?
Zhou J. et al. 2020	+	n.a.	+	+	?	+	+
Zhang J. et al. 2021	+	n.a.	?	+	?	+	?
Zhao H. et al. 2021	?	n.a.	?	+	?	+	-

B

Study participation
Study attrition
Prognostic factor measurement
Outcome measurement
Study confounding
Statistical analysis reporting
Overall risk of bias

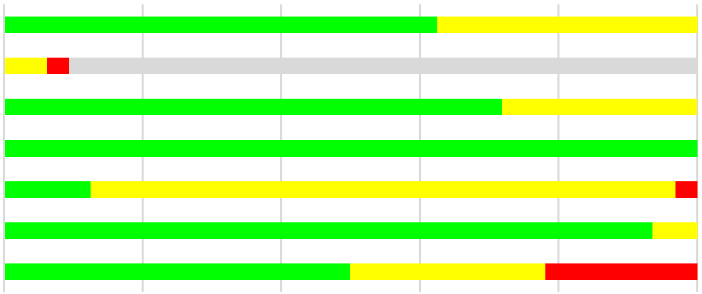

0% 20% 40% 60% 80% 100%

Low risk Moderate risk High risk Not applicable

Figure S12: Risk of bias assessment on study level [A] and across studies [B] comparing patients' gender in the repeatedly SARS-CoV-2 positive and non positive patients.

A

N.a. Not applicable
+ Low risk
? Moderate risk
– High risk

	Study participation	Study attrition	Prognostic factor measurement	Outcome measurement	Study confounding	Statistical analysis reporting	Overall risk of bias
Huang J. et al. 2020	+	n.a	+	+	?	+	+
Liu B. M. et al. 2020	?	n.a	+	+	?	+	?
Liu T. et al. 2020	+	n.a	+	+	?	+	+
Yang C. et al. 2020	+	n.a	+	+	+	+	+
Zou Y. et al. 2020	?	n.a	+	+	?	+	?
Liu H. et al. 2021	?	n.a.	+	+	?	+	?
Xu G. et al. 2021	?	n.a.	+	+	+	+	+

B

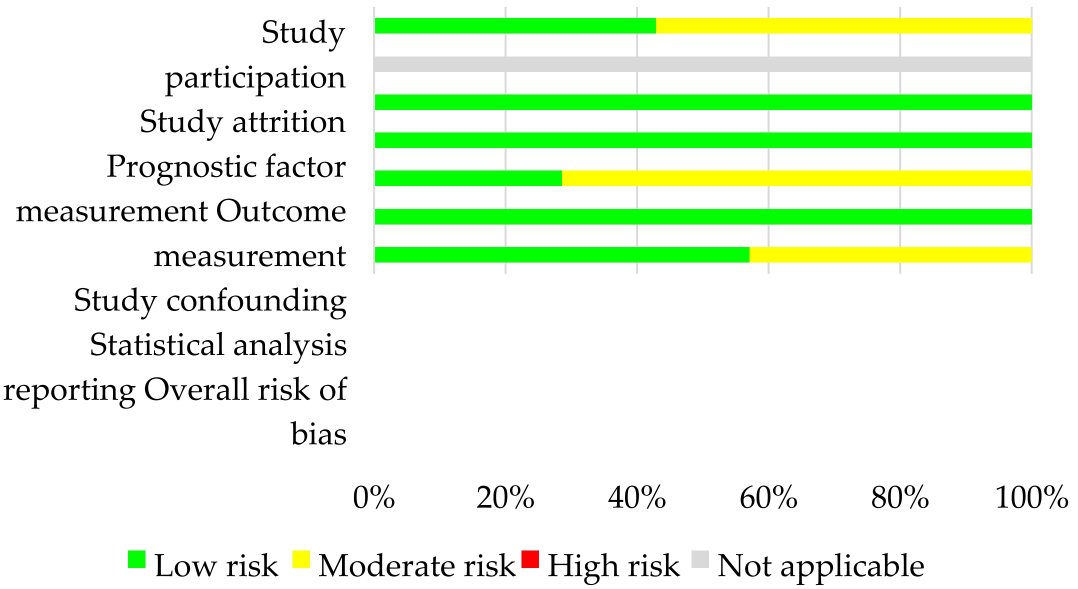

Figure S13: Risk of bias assessment on study level [A] and across studies [B] comparing IgG positivity in the repeatedly SARS-CoV-2 positive and non positive patients.

A

N.a. Not applicable<br>+ Low risk<br>? Moderate risk<br>– High risk	Study participation	Study attrition	Prognostic factor measurement	Outcome measurement	Study confounding	Statistical analysis reporting	Overall risk of bias
Huang J. et al. 2020	+	n.a	+	+	?	+	+
Liu B. M. et al. 2020	?	n.a	+	+	?	+	?
Liu T. et al. 2020	+	n.a	+	+	?	+	+
Yang C. et al. 2020	+	n.a	+	+	+	+	+
Zou Y. et al. 2020	?	n.a	+	+	?	+	?
Liu H. et al. 2021	?	n.a.	+	+	?	+	?
Xu G. et al. 2021	?	n.a.	+	+	+	+	+

B

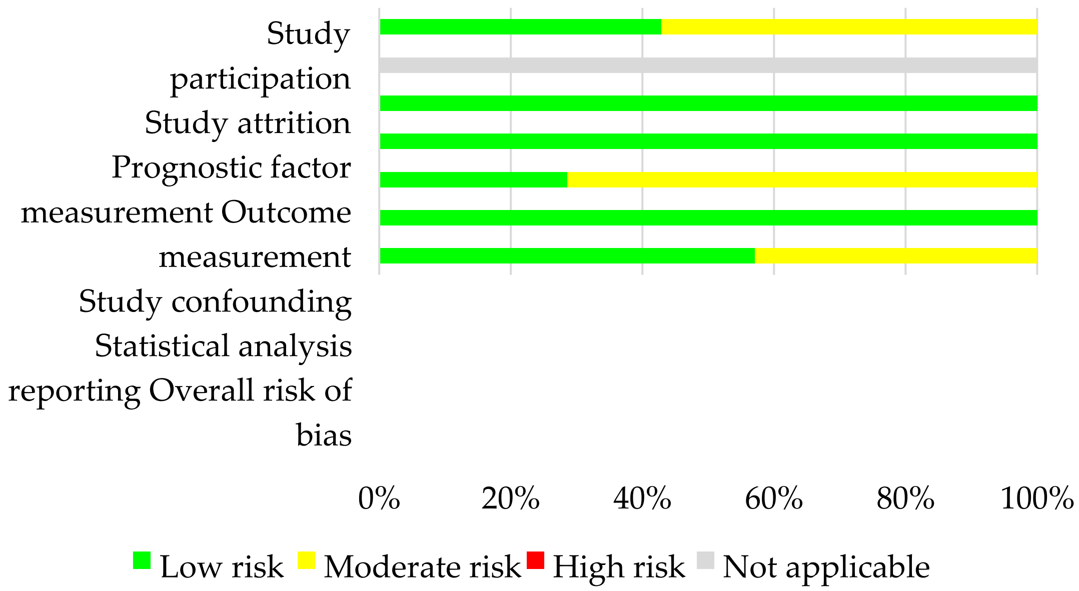

Figure S14: Risk of bias assessment on study level [A] and across studies [B] comparing IgM positivity in the repeatedly SARS-CoV-2 positive and non positive patients.

A

N.a. Not applicable; + Low risk; ? Moderate risk; – High risk	Study participation	Study attrition	Prognostic factor measurement	Outcome measurement	Study confounding	Statistical analysis reporting	Overall risk of bias
Hu R. et al. 2020	+	n.a	+	+	?	+	+
Yuan B. et al. 2020	+	n.a	+	+	-	?	-
An J. et al. 2020	+	n.a	+	+	?	+	+
Chen S. L. et al. 2020	+	n.a	+	+	?	+	+
Du H. et al. 2020	+	n.a	+	+	?	+	+
He S. et al. 2020	+	n.a	+	+	+	+	+
Huang J. et al. 2020	+	n.a	+	+	?	+	+
Landi F. et al. 2020	+	n.a	+	+	?	+	+
Shui T.J. et al. 2020	+	n.a	+	+	?	+	+
Yan N. et al. 2020	+	n.a	?	+	?	+	?
Yang C. et al. 2020	+	n.a	+	+	+	+	+
Zheng J. et al. 2020	?	?	+	+	?	+	-
Zou Y. et al. 2020	?	n.a	+	+	?	+	?
Ao Z. et. al. 2021	+	n.a.	?	+	+	+	+
Hong L. et al. 2021	+	-	+	+	?	+	-
Liu H. et al. 2021	?	n.a.	+	+	?	+	?
Liu J. et al. 2021	?	n.a.	?	+	+	+	?
Zhou J. et al. 2020	+	n.a.	+	+	?	+	+
Zhang J. et al. 2021	+	n.a.	?	+	?	+	?
Zhao H. et al. 2021	?	n.a.	?	+	?	+	-

B

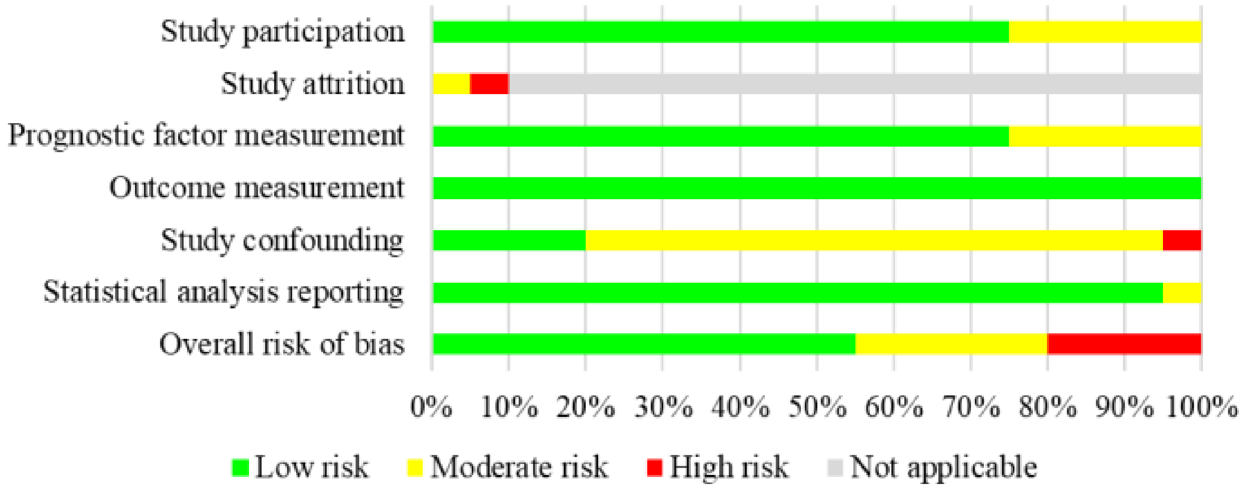

Figure S15: Risk of bias assessment on study level [A] and across studies [B] comparing the proportion of severe cases in the first infection in repeatedly SARS-CoV-2 positive and non positive patients.

A

N.a. Not applicable; + Low risk; ? Moderate risk; – High risk	Study participation	Study attrition	Prognostic factor measurement	Outcome measurement	Study confounding	Statistical analysis reporting	Overall risk of bias
An J. et al. 2020	+	n.a	+	+	?	+	+
He S. et al. 2020	+	n.a	+	+	+	+	+
Huang J. et al. 2020	+	n.a	+	+	?	+	+
Shui T.J. et al. 2020	+	n.a	+	+	?	+	+
Yan N. et al. 2020	+	n.a	?	+	?	+	?
Yang C. et al. 2020	+	n.a	+	+	+	+	+
Zheng J. et al. 2020	?	?	+	+	?	+	-
Zou Y. et al. 2020	?	n.a	+	+	?	+	?
Yang Z. et al. 2021	?	n.a.	+	+	?	+	?
Zhao H. et al. 2021	?	n.a.	?	+	?	+	-

B

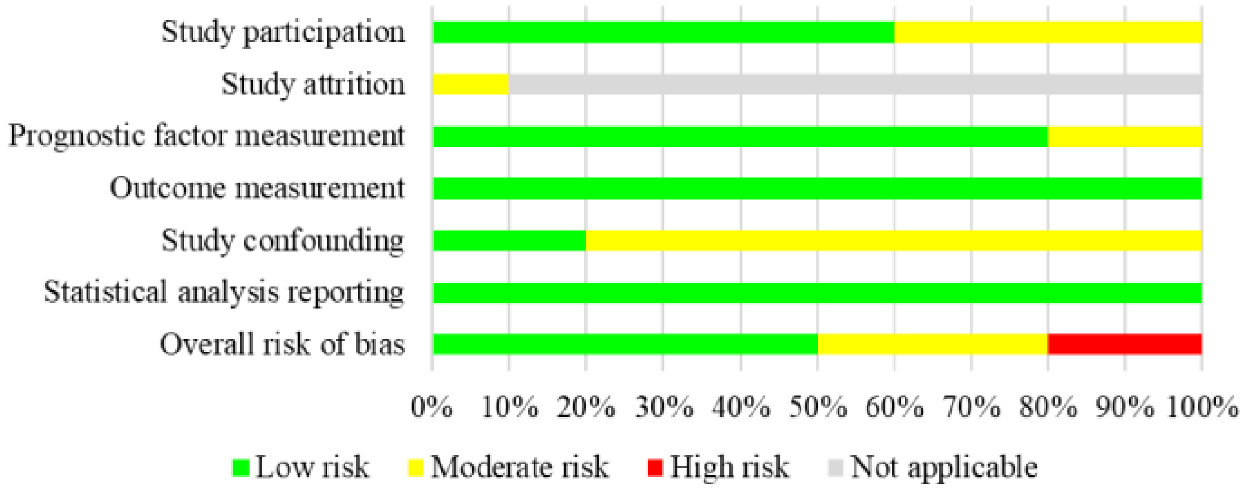

Figure S16: Risk of bias assessment on study level [A] and across studies [B] comparing the proportion of mild cases in the first infection in repeatedly SARS-CoV-2 positive and non positive patients.

A

N.a. Not applicable
+ Low risk
? Moderate risk
– High risk

	Study participation	Study attrition	Prognostic factor measurement	Outcome measurement	Study confounding	Statistical analysis reporting	Overall risk of bias
Yuan B. et al. 2020	+	n.a	+	+	-	?	-
Chen L.Z. et al. 2020	+	n.a	+	+	?	+	+
Chen S. L. et al. 2020	+	n.a	+	+	?	+	+
Du H. et al. 2020	+	n.a	+	+	?	+	+
He S. et al. 2020	+	n.a	+	+	+	+	+
Hu F. et al. 2020	?	n.a	?	+	?	+	-
Landi F. et al. 2020	+	n.a	+	+	?	+	+
Liu C. et al. 2020	?	n.a	?	+	?	?	-
Lu J. et al. 2020	+	n.a	+	+	?	+	+
Yan N. et al. 2020	+	n.a	?	+	?	+	?
Yang C. et al. 2020	+	n.a	+	+	+	+	+
Ye H. et al. 2020	+	n.a	+	+	?	+	+
Zheng J. et al. 2020	?	?	+	+	?	+	-
Zhu H. et el. 2020	?	n.a	+	+	?	+	?
Ao Z. et. al. 2021	+	n.a.	?	+	+	+	+
Hong L. et al. 2021	+	-	+	+	?	+	-
Liu H. et al. 2021	?	n.a.	+	+	?	+	?
Yang Z. et al. 2021	?	n.a.	+	+	?	+	?
Zhou J. et al. 2020	+	n.a.	+	+	?	+	+
Zhang J. et al. 2021	+	n.a.	?	+	?	+	?

B

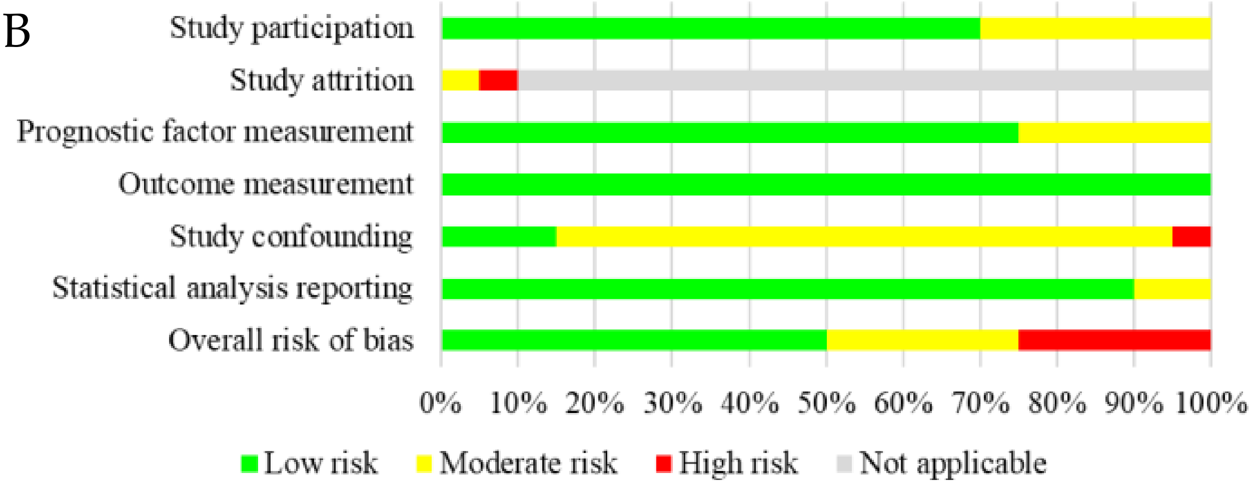

Figure S17: Risk of bias assessment on study level [A] and across studies [B] comparing length of hospital stay during the first infection in repeatedly SARS-CoV-2 positive and non positive patients.

A

N.a. Not applicable
+ Low risk
? Moderate risk
– High risk

	Study participation	Study attrition	Prognostic factor measurement	Outcome measurement	Study confounding	Statistical analysis reporting	Overall risk of bias
Chen L.Z. et al. 2020	+	*n.a*	+	+	?	+	+
Huang J. et al. 2020	+	*n.a*	+	+	?	+	+
Landi F. et al. 2020	+	*n.a*	+	+	?	+	+
Zhu, H. et el. 2020	?	*n.a*	+	+	?	+	?
Hong L. et al. 2021	+	-	+	+	?	+	-
Zhao H. et al. 2021	?	n.a.	?	+	?	+	-

B

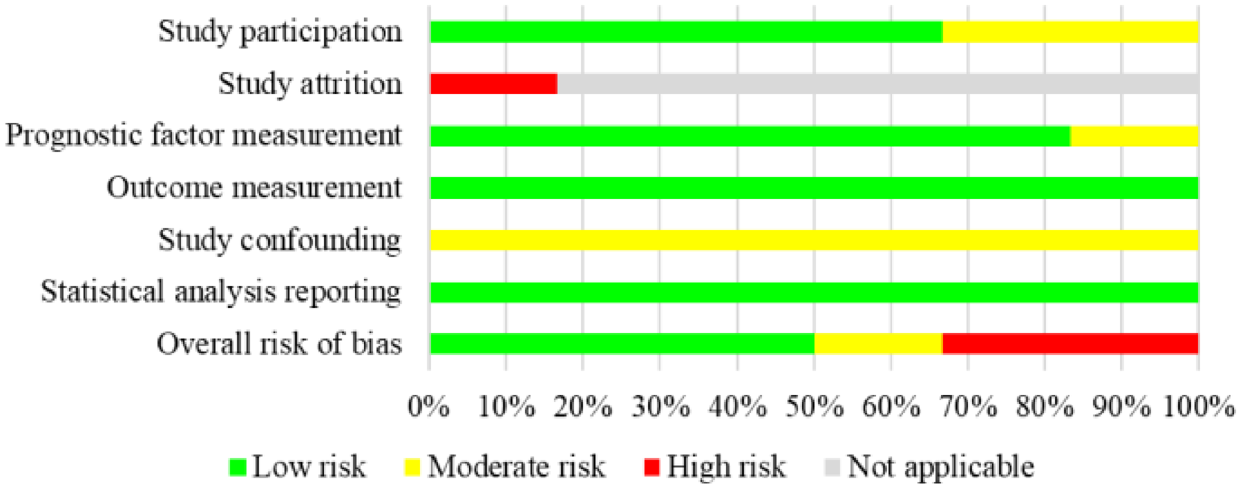

Figure S18: Risk of bias assessment on study level [A] and across studies [B] comparing body mass index (BMI) in repeatedly SARS-CoV-2 positive and non positive patients.
